# The changing vaccine landscape: rates of COVID-19 vaccine acceptance and hesitancy in young adults during vaccine rollout

**DOI:** 10.1177/17579139221094750

**Published:** 2022-05-15

**Authors:** H Knight, R Jia, K Ayling, H Blake, JR Morling, AM Villalon, J Corner, C Denning, J Ball, K Bolton, G Figueredo, D Morris, P Tighe, K Vedhara

**Affiliations:** School of Medicine, University of Nottingham, Nottingham NG7 2RD, UK; School of Medicine, University of Nottingham, Nottingham, UK; School of Medicine, University of Nottingham, UK; School of Health Sciences, University of Nottingham, Nottingham, UK; NIHR Nottingham Biomedical Research Centre, Nottingham, UK; School of Medicine, University of Nottingham, Nottingham, UK; NIHR Nottingham Biomedical Research Centre, Nottingham, UK; Faculty of Engineering, University of Nottingham, Nottingham, UK; University Executive Board, University of Nottingham, Nottingham, UK; Biodiscovery Institute, University Park, University of Nottingham, Nottingham, UK; Biodiscovery Institute, University Park, University of Nottingham, Nottingham, UK; School of Mathematical Sciences, University of Nottingham, Nottingham, UK; School of Computer Sciences, University of Nottingham, Nottingham, UK; Faculty of Engineering, University of Nottingham, Nottingham, UK; School of Life Sciences, University of Nottingham, Nottingham, UK; School of Medicine, University of Nottingham, Nottingham, UK

**Keywords:** COVID-19, vaccine hesitancy, public health

## Abstract

**Aims:**

Development and rollout of vaccines offers the best opportunity for population protection against the SARS-CoV-2 (COVID-19) virus. However, hesitancy towards the vaccines might impede successful uptake in the United Kingdom, particularly in young adults who demonstrate the highest rates of hesitancy. This prospective study explored COVID-19 vaccine hesitancy in young adults and whether the reasons behind these attitudes changed during the initial stages of the United Kingdom’s vaccine rollout.

**Method:**

Data on vaccination intention were collected from a British university student cohort at three time points: October 2020, February 2021, and March 2021. This online survey included items on intention to receive a vaccine and a free-text response for the reasons behind this intention. Cochran’s Q tests examined changes in rates of hesitancy and acceptance over time and free-text responses were analysed thematically.

**Results:**

At baseline, 893 students provided data, with 476 participants completing all three time points. Hesitancy declined over time, with 29.4% of participants expressing hesitancy at baseline, reducing to 9.1% at wave 2 and 5.9% at wave 3. The most commonly endorsed themes for those willing to accept a vaccine were self-protection against COVID-19 and pro-social reasons, including protecting the population or unspecific others, and ending the pandemic/returning to normal life. The most commonly endorsed hesitancy themes related to ‘confidence’ in the vaccines and potential personal risk, including insufficient testing/scientific evidence, concern about side effects, and long-term effects. These reasons remained the most commonly endorsed at both waves 2 and 3.

**Conclusions:**

While a decline in hesitancy was observed over time, the key reasons behind both vaccine acceptance and hesitancy remained consistent. Reasons behind hesitancy aligned with those of the general public, providing support for the use of generalist interventions. Pro-social reasons frequently underpinned vaccine acceptance, so cohort-specific interventions targeting those factors may be of benefit.

## Introduction

The ongoing COVID-19 pandemic has resulted in the deaths of over six million people thus far (Coronavirus Worldometer) and substantial social restrictions worldwide. Development of COVID-19 vaccines rapidly became the focus of global research, offering an important mechanism for controlling transmission. In December 2020, the UK initiated a rapid COVID-19 vaccine programme, resulting in the vaccination of almost 50 million adults to date (3 May 2022^1^). Although uptake of the vaccines in prioritised populations has been relatively high, general mistrust of the vaccines and concerns about potential side effects may impede the UK’s ability to achieve population immunity through vaccination.

Hesitancy towards receiving a COVID-19 vaccine has declined during the UK’s vaccine rollout.^2^ However, data continue to highlight a discrepancy between older and younger adults. As of April 2022, rates of complete (two dose) vaccine uptake in young adults ranged from 64.6% (ages 25–under 30) to 64.1% (ages 18–under 20),^3^ despite the vaccine being readily available to these age groups and the introduction of vaccination requirements for some social events and travel. In contrast, over 90% of adults aged 60+ have already received a COVID-19 vaccine.^3^ Young adults play an integral role in population immunity,^4^ and rates remain significantly lower than that of middle-aged and older adults. Attitudes towards vaccine safety, their importance and their effectiveness are consistently associated with vaccine uptake.^5^ Tracking changes in attitudes towards the COVID-19 vaccines is important to ensure public health interventions continue to target key concerns. However, research has predominantly focused on the general population, rather than the concerns unique to young adults. We report here a prospective study exploring COVID-19 vaccine hesitancy and acceptance in young adults, and whether the reasons behind these attitudes changed during the initial stages of the UK’s vaccine rollout (Feb-March 2021).

## Method

As part of a longitudinal university student cohort study, data on COVID-19 vaccination intention were collected at three time points: 5 October 2020 to 1 November 2020 (before vaccination rollout; baseline), 1 February 2021 to 26 February 2021 (during rollout; wave 2), 10 March 2021 to 26 March 2021 (during rollout; wave 3). This included vaccination intention (‘If you were offered a COVID-19 vaccine, would you take it?’; response options: Yes/No/Unsure) and a free-text response to elaborate on the reasons behind this intention. We defined vaccine hesitancy as participants providing ‘No’ or ‘Unsure’ responses. An option ‘I have already had a COVID-19 vaccine’ was added at wave 3. Ethical approval was received from the University of Nottingham Faculty of Health Sciences Research Ethics Committee.

## Statistical and Content Analysis

Independent *t*-test and chi-square tests compared differences in demographics and vaccine attitudes at baseline between completers and non-completers (did not complete all three surveys). Cochran’s Q tests examined changes in rates of hesitancy and acceptance over time. Free-text responses were analysed using inductive content analysis; responses were coded into themes and the frequency of common themes was subsequently quantified. If free-text responses contained multiple themes, each theme was coded separately. Follow-up with non-completers was not conducted.

## Results

### Cohort characteristics

Participant characteristics are summarised in Table 1. In total, 893 participants (mean age = 21 years) provided baseline data (October 2020). The cohort was predominantly undergraduate (*n* = 789, 88%), 63% female (*n* = 556), and 66% white British (*n* = 589), aligning with gender and ethnicity characteristics of undergraduates in British higher education (57% female, 74% White; 2019–2020). 540 students completed the wave 2 survey (February 2021), with 476 providing data at all time points. Students who discontinued the study were more likely to be male (χ^2^ = 28.7, *p* < .001) and from an ethnic minority background (χ^2^ = 7.6, *p* = .006). No difference in baseline vaccine hesitancy was found between completers and non-completers (*p* = .70; see Supplemental Table 1).

**Table 1:** Demographic characteristics and vaccine attitudes.

	Baseline	Wave 2	Wave 3
	*n* (%)	*n* (%)	*n* (%)
Total *N* completers	893 (100%)	540 (100%)	476 (100%)
Age (mean, SD)	20.7 (3.4)		
Gender	889 (99.6%)		
Male	333 (37.5%)		
Female	556 (62.5%)		
Ethnicity	893 (100%)		
White – British, Irish, other	589 (66.1%)		
Ethnic minority background	303 (33.9%)		
Asian/Asian British – Indian, Pakistani, Bangladeshi, other	102 (11.4%)		
Black/Black British – Caribbean, African, other	43 (4.8%)		
Chinese/Chinese British	82 (9.2%)		
Middle Eastern/Middle Eastern British – Arab, Turkish, other	12 (1.3%)		
Mixed race	34 (3.9%)		
Other ethnic group	20 (2.2%)		
Prefer not to say	10 (1.1%)		
Level of study	893 (100%)		
Undergraduate	789 (88.4%)		
Postgraduate	94 (10.5%)		
Other	10 (1.1%)		
Vaccine attitude responses (‘If offered a vaccine, would you take it?’)^a^	893 (100%)	540 (100%)	476 (100%)
Yes	631 (70.7%)	491 (90.9%)	334 (70.2%)
No	56 (6.3%)	15 (2.8%)	7 (1.5%)
Unsure	206 (23.1%)	34 (6.3%)	21 (4.4%)
Already had a COVID-19 vaccine	N/A	N/A	114 (24.0%)
Participants providing codable free-text responses^b^	699 (78.3%)	369 (68.3%)	226 (47.5%)
One code	487 (69.7%)	249 (67.5%)	143 (63.3%)
Two codes	190 (27.2%)	102 (27.6%)	72 (31.9%)
Three or more codes	22 (3.1%)	18 (4.9%)	11 (4.8%)

a Total *N* completing each time point.

b *n* providing codable responses out of total *N* completing time point.

### Vaccine hesitancy and acceptance

At baseline, 29.4% of participants (*n* = 262) were hesitant about receiving a vaccine, reducing to 9.1% (*n* = 49) at wave 2 and 5.9% (n = 28) at wave 3, with *n* = 114 (24%) participants having already received a vaccine (Supplemental Figure 1). This declining pattern of hesitancy was also observed in participants completing all three surveys (*n* = 476), (baseline to wave 2: Cochran’s Q = 78.7, *p* < .001; waves 2 to 3: Cochran’s Q = 4.17, *p* = .041; baseline to wave 3: Cochran’s Q = 96.59, *p* < .001.

### Reasons for hesitancy and acceptance

At baseline, 699 participants (78.3%) provided free-text responses (*n* = 205 for hesitancy, *n* = 494 for acceptance) allowing coding of 935 unique responses; 316 responses related to hesitancy themes and 619 related to acceptance (see Table 1). The most commonly endorsed themes for those willing to accept a vaccine were ‘self-protection against COVID-19’ (40% of responses), to ‘protect the population or unspecific others, and control the virus’ (35%), to ‘end the pandemic and return to normal life’ (12%), and ‘protect specific others’ (6%). The most commonly endorsed hesitancy themes related to ‘confidence’ in the vaccines and potential personal risk, including insufficient testing/scientific evidence (20%), concern about side effects (18%), long-term effects (13%), speed of the vaccines’ development (10%), general safety issues (9%), and general effectiveness (9%). Other infrequent responses related to lack of knowledge, a belief in existing protection or being at low risk of contracting COVID-19.

At wave 2, 73% (*n* = 393) of the sample provided free-text responses (*n* = 32 for hesitancy, *n* = 361 for acceptance). At wave 3, 55% (*n* = 260) provided free-text responses (*n* = 17 for hesitancy, *n* = 243 for acceptance). The primary themes identified at baseline remained the most commonly endorsed across waves 2 and 3 for both hesitancy and acceptance, with no new themes emerging (for detailed breakdown, see Supplemental Figures 2 and 3, and Supplemental Tables 2 and 3).

## Discussion

Vaccine hesitancy may serve as a limiting factor in national and global attempts to control the COVID-19 pandemic. Young adults play an integral role in the effectiveness of vaccination programmes; understanding the reasons for hesitancy and acceptance is key to successful rollout.^4^ This short report illustrates, reassuringly, that in a student population, rates of hesitancy significantly declined during the vaccine rollout. Despite the short period of time between waves 2 and 3, a continued decline in hesitancy was still observed. Our work echoes the final ONS ‘Coronavirus and vaccine hesitancy’ report (9 August 2021) which demonstrated a substantial decrease in young adults’ vaccine hesitancy over the first two years of the pandemic and that the majority of young adults were supportive of receiving a COVID-19 vaccine as of August 2021.^2^ Rollout of the vaccines has allowed the accrual of evidence on safety and long-term effectiveness, along with observable declines in infection and death rates. As baseline hesitancy themes were strongly tied to confidence in the vaccines and perceived personal risk, vaccine rollout has perhaps allayed these concerns.

Interestingly, while changes to the rate of hesitancy were observed over time, the key reasons behind acceptance and hesitancy remained consistent. Recent research shows beliefs about the collective importance of vaccination, vaccine efficacy, concern about side effects, and speed of vaccine development are key to targeting COVID-19 vaccine hesitancy,^6^ with these factors explaining a substantial amount of variance (86%) in a large sample of British adults.^7^ Our data suggest those who continue to experience hesitancy do so for the same reasons as those who were hesitant prior to the vaccines’ rollout. Similarly, the primary reasons behind vaccine acceptance did not change over time, although overall rates of acceptance increased. Taken together, these findings suggest that the reasons for both hesitancy and acceptance in young adults are similar to those found in the general public, and that while the number of people who appear to have these concerns is declining, the core reasons for hesitancy remain. As this age group, and those under the age of 18, are currently eligible for COVID-19 vaccination in the UK, future work should continue to monitor changes to hesitancy, particularly given the observed discrepancy between reported willingness to accept a vaccine and uptake in this age group.^2,3^ Given the alignment of these concerns with those of the wider population, evidence-based interventions targeting vaccine hesitancy in the general public^6^ might prove helpful.

Our findings also demonstrate young adults are motivated to accept a vaccine for pro-social reasons, although this form of messaging framing has been largely underutilised to date. Social media has been used to improve uptake of other vaccines,^8^ with younger adults frequently relying on social media for health information.^9^ However, the potential for misinformation to fuel hesitancy concerns on these platforms is high, particularly as government-produced posts about COVID-19 only account for a small fraction (approx. 11%) of those available on social media outlets.^10^ We must therefore ensure public health campaigns which utilise these communication pathways should do so effectively.

While our findings provide a step towards understanding hesitancy in young adults, limitations are noted. Our sample comprised university students who may demonstrate greater health literacy and lower levels of vaccine hesitancy than the general population, perhaps explaining the higher rate of vaccine acceptance (94.1%) found in this sample compared with ONS data collected during the same time period (87%).^2^ This limits the generalisability of our findings to young adults more broadly. Over the course of the study, 417 participants were lost to follow-up by time point 3. Unfortunately, we were unable to follow-up with those who did not complete all time points; however, our data identified that non-responders were more likely to be from an ethnic minority background. Recent research shows individuals from certain ethnic minorities are more likely to demonstrate higher rates of vaccine hesitancy and report concerns that vary from their White counterparts.^11^ Future research should examine contributors to vaccine hesitancy in young adults outside of tertiary education, with a focus on exploring the concerns of those from ethnic minorities and diverse socioeconomic backgrounds.

## Supplemental Material

sj-docx-1-rsh-10.1177_17579139221094750 – Supplemental material for The changing vaccine landscape: rates of COVID-19 vaccine acceptance and hesitancy in young adults during vaccine rollout Supplemental material, sj-docx-1-rsh-10.1177_17579139221094750 for The changing vaccine landscape: rates of COVID-19 vaccine acceptance and hesitancy in young adults during vaccine rollout by H Knight, R Jia, K Ayling, H Blake, JR Morling, AM Villalon, J Corner, C Denning, J Ball, K Bolton, G Figueredo, D Morris, P Tighe and K Vedhara in Perspectives in Public Health
