# Supplementary material for: The changing vaccine landscape: rates of COVID-19 vaccine acceptance and hesitancy in young adults during vaccine rollout
Source: Perspect Public Health. 2022 May 15;143(4):220–4. doi: 10.1177/17579139221094750 (PMC10467000; doi:10.1177/17579139221094750)
Supplement: sj-docx-1-rsh-10.1177_17579139221094750 – Supplemental material for The changing vaccine landscape: rates of COVID-19 vaccine acceptance and hesitancy in young adults during vaccine rollout [file sj-docx-1-rsh-10.1177_17579139221094750.docx]

Supplementary Figure 1. Change in rates of COVID-19 vaccine hesitancy and acceptance amongst completers of all survey time points

Supplementary Figure 2. Frequency of endorsed themes relating to vaccine hesitancy at baseline, wave 2 and wave 3

Supplementary Figure 3. Frequency of endorsed themes relating to vaccine acceptance at baseline, wave 2 and wave 3

Supplementary Table 1. Demographic characteristics by completion status

|  | All participants | Completers^a^ | Non-completers^b^ |
| --- | --- | --- | --- |
|  | n (%) | n (%) | n (%) |
| N | 893 (100%) | 476 (53.3%) | 417 (46.7%) |
| Age (mean, SD) | 20.7 (3.4) | 20.8 (3.7) | 20.6 (3.0) |
| Gender* | 889 (99.6%) | 474 (99.6%) | 415 (99.5%) |
| Male | 333 (37.5%) | 139 (29.3%) | 194 (46.8%) |
| Female | 556 (62.5%) | 335 (70.7%) | 221 (53.3%) |
| Ethnicity* | 893 (100%) | 476 (100%) | 417 (100%) |
| White – British, Irish, other | 589 (66.1%) | 334 (70.2%) | 256 (61.4%) |
| Ethnic minority background | 303 (33.9%) | 142 (29.8%) | 161 (38.6%) |
| Level of study* | 893 (100%) | 421 (88.4%) | 378 (90.6%) |
| Undergraduate | 789 (88.4%) | 409 (97.2%) | 357 (94.4%) |
| Postgraduate | 94 (10.5%) | 11 (2.6%) | 12 (3.2%) |
| Other | 10 (1.1%) | 1 (0.2%) | 9 (2.4%) |

Supplementary comparison of demographic characteristics between those who completed all 3 timepoints, and those who did not. ^a^Provided data across all three timepoints, as a total percentage of those providing baseline data; ^b^ Provided data at baseline only or baseline and wave 2, as a total percentage of those providing baseline data; *Significant differences between completers and non-completers.

Supplementary Table 2. Common themes behind vaccine hesitancy

| Themes | Count | Examples of free text responses |
| --- | --- | --- |
| Concerns about unknown long-term effects | Baseline: 41  Wave 2: 9  Wave 3:4 | “It’s a new vaccine with no idea what the long term effects are”  “Any vaccine that hasn't been around for years and years I am slightly apprehensive about - I want to know any long term effects of a vaccine (even if small risk)” |
| Concerns about side effects | Baseline: 58  Wave 2: 9  Wave 3: 3 | “The uncertainty of the side effects scares me”  “Slightly scared of the undiscovered side effects that may occur” |
| Concerns there is an insufficient testing/evidence base | Baseline: 62  Wave 2: 3  Wave 3: 3 | “It’s new and just unknown at the moment”  “I would want lots of scientific data and tests before I put anything in my body” |
| Concerns the development of the vaccine has been rushed | Baseline: 31  Wave 2: 1  Wave 3: 1 | “Concerned about the short time it has taken to develop the vaccine, as opposed to the usual timescale for vaccines.”  “The rushed trials decreases safety long term” |
| Concerns about the safety of the vaccine (but not explicitly side effects) | Baseline: 28  Wave 2: 3  Wave 3: 0 | “I do not think it’s safe” |
| Unsure about vaccine effectiveness | Baseline: 27  Wave 2: 2  Wave 3: 0 | “I don’t believe that a vaccine would stop you getting COVID-19” |
| Lack of trust in the manufacturer/government/scientists etc. | Baseline: 14  Wave 2: 1  Wave 3: 3 | “Don’t trust the government”  “So long as it was approved by the appropriate organisations and not just the government going against expert opinion” |
| Believe they are in good health / Their body can fight off the virus | Baseline: 14  Wave 2: 4  Wave 3: 2 | “I think as a young person I am better off getting covid 19 as it has been shown that young people experience next to little symptoms”  “Because I don't need to. I won't be affected by the virus” |
| Other people need it more | Baseline: 11  Wave 2: 5  Wave 3: 0 | “I would rather someone more at risk have it than me!”  “There are more deserving people who need it more than me” |
| Other reasons (e.g. lack of knowledge, belief in existing protection, perception of being at low risk) | Baseline: 25  Wave 2: 4  Wave 3: 4 | “With my underlying health conditions, unsure if I would be allowed as I cannot have the live vaccines”  “I'd like to know more about it before making a decision” |

Supplementary Table 3. Common themes behind vaccine acceptance.

| Themes | Count | Examples of free text responses |
| --- | --- | --- |
| Self-protection | Baseline: 247  Wave 2: 164  Wave 3: 120 | “I'd rather be immune to Covid 19 than not”  “I'm at additional risk if get COVID and I don't want to worry about that any more.” |
| Protect specific others (e.g., family, friends, colleagues etc.) | Baseline: 36  Wave 2: 48  Wave 3: 35 | “I’d want to be protected from the virus so I’m able to go home without risking my family”  “So that I don’t get it in the future” |
| Protect the population/non-specific others and control the virus | Baseline: 215  Wave 2: 149  Wave 3: 98 | “To contribute to herd immunity”  “I would take it to prevent transmission to those more vulnerable than me.” |
| Hope to end the pandemic/ wish for normal life | Baseline: 77  Wave 2: 90  Wave 3: 61 | “So I can get back to normal life asap”  “To end the pandemic” |
| Non-specific pro-vaccine/pro-science statement | Baseline: 34  Wave 2: 19  Wave 3: 11 | “I believe in science”  “Vaccinations are essential to stopping the spread of disease” |
| Civil duty/Requirement | Baseline: 7  Wave 2: 7  Wave 3: 6 | “To do my part for the NHS” |
| Confidence in SARS-Cov-2 vaccine | Baseline: 3  Wave 2: 19  Wave 3: 11 | “I have trust in the vaccine approval system.” |
